# The influence law of concrete aggregate particle size on acoustic emission wave attenuation

**DOI:** 10.1038/s41598-021-02234-x

**Published:** 2021-11-22

**Authors:** Xin Wu, Qiao Yan, Ahmadreza Hedayat, Xuemei Wang

**Affiliations:** 1 College of Engineering, Sichuan Normal University, Chengdu, 610101 Sichuan China; 2 Department of Civil and Environmental Engineering, Colorado School of Mines, Golden, CO 80401 USA

**Keywords:** Civil engineering, Mechanical properties, Engineering

## Abstract

Elastic waves have different attenuation laws when propagating in various materials, which is one of the important challenges in the application of non-destructive testing methods, such as acoustic emission (AE) technology in geotechnical engineering. The study presented in this paper investigated the influence mechanism of concrete composition materials and parameters on the propagation law of elastic waves using concrete specimens produced in six different particle sizes of sand or gravel. The burst AE signal was generated through the lead-breaking experiment, and ceramic piezoelectric sensors were used to record the signal waveform at different propagation distances. Through parameter analysis, spectrum analysis, and pattern recognition techniques, the influence of the concrete aggregate particle size on AE wave propagation and attenuation was revealed. The results show that the attenuation of elastic wave amplitude, energy spectral density, and frequency all were positively correlated with the aggregate particle size, and the elastic wave spectrum center of gravity generally decreased with the propagation distance. The ring count gradually decreased with the propagation distance, and the specimens with a larger aggregate particle size underwent a relatively faster ring count attenuation rate. The rise time increased rapidly with the propagation of the elastic wave, and the specimens with a larger aggregate particle size experienced a relatively rapid increase in rise time. In addition, in the feature spaces of ring count-amplitude and rise time–amplitude, the size of aggregate has an obvious influence on the distribution of these feature vector.

## Introduction

The phenomenon that a local source quickly releases energy in material to generate a transient elastic wave is called acoustic emission (AE), which is essentially an elastic wave with various frequencies and mode^1,2^. Acoustic emission, as an important nondestructive testing method, has many advantages, such as high sensitivity, timely response, and full-cycle real-time monitoring, and has broad application prospects in the field of engineering safety monitoring. Elastic waves have different attenuation laws when they propagate in various materials. This is one of the most important challenges in the application of non-destructive testing methods, such as AE in geotechnical engineering. Some scholars have carried out a large number of experimental studies to analyze the influence of various parameters of rock materials on AE signal attenuation, including water saturation^3^, confining pressure, the crack angle, and the number of cracks^4^ and so on. Not limited to rock materials, the propagation distance and attenuation law of AE signal in sandstone^5^ and coal^6,7^ have also been studied, and some preliminary conclusions are obtained. In other common building materials, the attenuation of AE signal also has some research results in recently. For instance, the propagation law of AE in metal bars in concrete^8^, AE propagation in steel pipes^9^, and the attenuation changes of AE signal propagation induced by damage in some composites are also used to study the damage of materials^10,11^. Techniques based on acoustic emission signal feature analysis, spectrum analysis and pattern recognition are widely used in feature extraction and analysis of attenuated signals^12–14^. In addition, some scholars analyze the attenuation mechanism of seismic wave propagation by establishing wave equation^15^ and mathematical model, such as Modified Omori Law (MOL)^16^. Dispersion and attenuation of elastic waves in anisotropic porous media^17–19^ has also received attention. Further, there are Literatures based on numerical modeling to study the influence of pore structure on AE signal attenuation^20^. In addition, not limited to the above research results, the attenuation law of AE signals in various geological and building materials has been studied^21–23^.

Briefly, a great deal of research has been conducted on the propagation and attenuation laws of elastic waves in various materials such as rocks. However, as a common building material, the attenuation law of elastic wave in concrete is not deeply studied. Very little research has addressed the characteristics of concrete materials, such as aggregate particle size, and the influence of elastic wave propagation combined even though they are important considerations that are known to affect the accuracy of AE, ultrasonic, and other non-destructive testing methods. To address this knowledge gap, this paper, therefore, presents the results of such a study investigating the influence of aggregate particle size on elastic wave attenuation using different sizes of sand or gravel as the aggregate to make the concrete samples and AE monitoring as the research method to obtain the law of elastic wave attenuation through data analysis.

## AE experiments

When the peak stress exceeds the local strength of the material, the material will crack and deform from that stress point to release energy, and the position of this point is the AE source. The elastic wave from the AE source will pass through the interface of different materials and will be received by sensors placed on the surface of the material. In the study presented in this paper, the burst signal generated by the lead-break test was used to simulate the AE source to study the attenuation law of elastic waves in concrete materials.

### Preparation of specimens

To determine the influence of different aggregate particle sizes on the attenuation of elastic waves, the continuous gradation of ordinary building natural sand and gravel was used as the raw material. The GZS-1 high frequency vibrating screen was used to screen out six size ranges of sand or gravel particles (0.5–1 mm, 1–2 mm, 2–5 mm, 5–10 mm, 10–15 mm, and 15–20 mm), which were used as the aggregate for the concrete specimens in this study. To obtain the influence law of the concrete aggregate particle size on the attenuation of elastic wave parameters, a higher aggregate mass ratio was selected within a reasonable range for the specimens. Several prefabrications were carried out, and the mass ratio of the sample was finally determined (i.e., cement:sand:water = 1:2:0.5).

**Figure 1:**
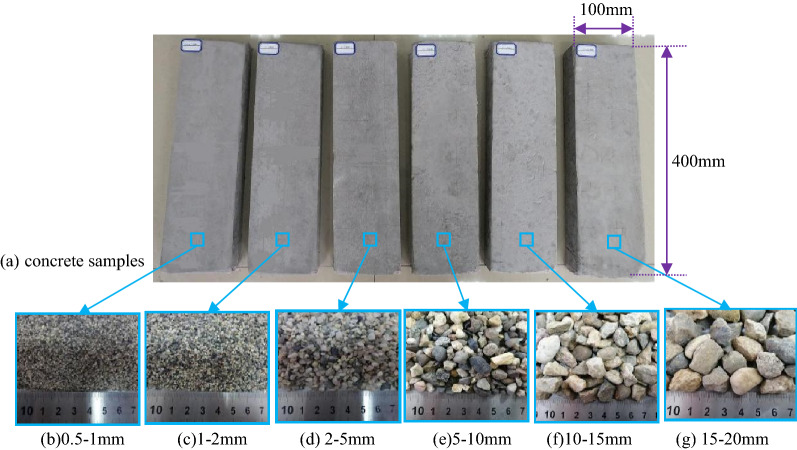
(**a**) Preparation of concrete samples. (**b**–**g**) The concrete samples with aggregate particle sizes.

The ingredients were poured into a mixing container according to the above mass ratio, mixed thoroughly and evenly, and poured into a special mold for the concrete test pieces with a size of 100 mm × 40 mm × 400 mm. Then, the mold was placed on the vibrating table and vibrated for 10 min to eliminate any air bubbles inside the sample and to improve the density of the sample. Finally, the mold was placed in a constant temperature and humidity curing box for 28 days, after which the sample was removed from the mold with a demolding gun. Figure 1, from left to right shows the concrete samples with aggregate particle sizes of 0.5–1 mm, 1–2 mm, 2–5 mm, 5–10 mm, 10–15 mm, and 15–20 mm. The samples were polished, and the flatness was controlled within ± 0.05 mm.

### Experiment methods

**Figure 2:**
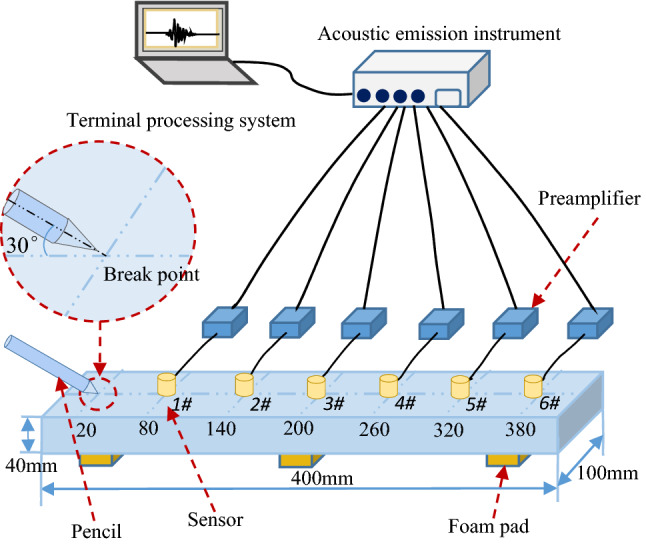
Test principle diagram (Drawing by WPS Office 2016, https://platform.wps.cn/).

The tests were conducted using a DS5-16B type AE tester, a USB3.0 interface that could support the 16-channel synchronous acquisition and continuously record and store 11.6 h of AE data. The sampling frequency selected for the tests was 2.5 MHz. Six RS-2A ceramic piezoelectric sensors with a frequency response range of 100–400 kHz and six 40 dB preamplifiers were used. The lead-breaking position and the sensor arrangement position were marked along the long axis of the center of the test piece; the lead-breaking position was set at 20 mm from the left edge; the 1–6# sensors were set at 80 mm, 140 mm, 200 mm, 260 mm, 320 mm, and 380 mm, as shown in Fig. 2.

Each sample was tested for lead-breaking four times. Petroleum jelly was used as the coupling agent between the ceramic piezoelectric sensor and the surface of the test piece to improve the efficiency of transduction and to reduce the loss and distortion of the signal.The lead-breaking test was carried out at an angle of 30° with an HB pencil refill of 0.5 mm in the plane of the test piece. The four test results under the same conditions were named Test 1, Test 2, Test 3, and Test 4. All the waveform data of the lead-breaking test process through the 1–6# sensors were collected and processed. Digital filters were designed according to the sensor parameters; and filtering, spectrum conversion, parameter statistics, and pattern recognition then were performed to analyze the influence mechanism of the aggregate particle size on the attenuation process of the elastic waves in the concrete specimens.

### Particle analysis

Although different grades of sand and gravel particles were obtained through physical screening tests, the distribution law of the grades within the same particle size range was still unknown. Therefore, to further analyze the true particle size distribution, the following six types of sand particles were analyzed based on the digital image analysis.

Due to the different color depths of the selected sand or gravel particles, the gravel particles first were given a color to improve the image recognition accuracy. Representative sand or gravel samples were selected in each size, and the sand grains were infiltrated with carbon ink and thoroughly dried before use. The air-dried sand particles then were distributed as evenly as possible within a certain sampling range (fine aggregate sampling range 5 cm × 5 cm and coarse aggregate sampling range 15 cm × 15 cm), while trying to maintain a certain gap between the particles to reduce identification errors. Then, the image acquisition was performed under uniform natural light; the acquired image was binarized; the superimposed particles were decomposed using a watershed algorithm. The particles were numbered according to the image; the equivalent diameter and area measurements were calculated; and finally, the particle distribution parameters were converted according to the above information as shown in Table 1.

**Table 1:** Particle size distribution based on digital image analysis.

Particle category	Particle size range	Grain grayscale	Particle identification chart	Particle size distribution (mm)
Fine aggregate 5 cm × 5 cm	0.5–1 mm			x = 0.937 σ = 0.186 x_min_ = 0.498 x_max_ = 1.541
	1–2 mm			x = 1.734 σ = 0.293 x_min_ = 1.187 x_max_ = 2.602
	2–5 mm			x = 3.381 σ = 0.664 x_min_ = 2.000 x_max_ = 5.347
Coarse aggregate 15 cm × 15 cm	5–10 mm			x = 6.527 σ = 1.193 x_min_ = 4.217 x_max_ = 10.503
	10–15 mm			x = 13.562 σ = 1.848 x_min_ = 9.636 x_max_ = 17.433
	15–20 mm			x = 17.209 σ = 1.763 x_min_ = 14.162 x_max_ = 20.991

As the particle size increased, the particle size distribution recognized by the particles was more consistent with the physical screening results. By collecting images after the artificial color was applied, the problem of indistinguishability between the white quartz particles and the white background color was avoided and a good binarization effect was achieved; but in the process of identifying small particles (e.g., 0.5–1 mm, 1–2 mm), the particle size recognized by the image was larger than the screening result, which may have been related to the formation of some larger cohesive particles in the coloring process. For the larger particles (2–5 mm, 5–10 mm, 10–15 mm, and 15–20 mm), the recognition effect was similar to the screening results so the measurement results approximately reflected the true particle distribution.

**Figure 3:**
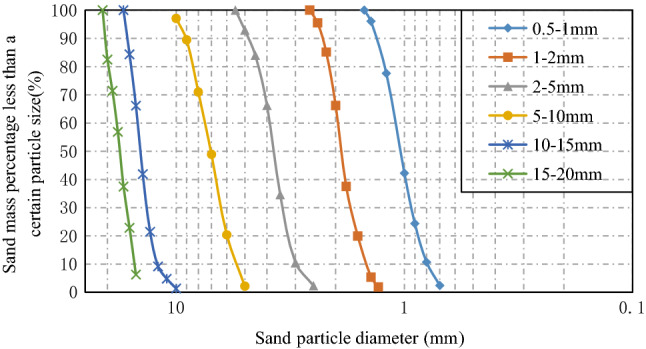
Gradation cumulative curve of sand particle size.

Since the density of the sand produced in the same stockyard and the same batch was similar, the sand was regarded as uniform density. Assuming the density of the sand body was $\rho$, the mass of the sand was volume × density (i.e., ${\text{m}} = V \times \rho$). On this basis, combined with the statistical information about the particle distribution, the.

particle gradation curve was drawn, as shown in Fig. 3. It can be seen that the distribution curve of the particle gradation cumulative curves of the various samples was steep, the slope was large, and the distribution of the sample particle size was relatively concentrated.

In engineering, the coefficient of uniformity, C_u_, and the coefficient of curvature, C_c_, generally are used to characterize the particle size distribution. The formula is:

1 $$\left\{ {\begin{array}{*{20}l} {C_{u} = \frac{{d_{60} }}{{d_{10} }}} \hfill \\ {C_{c} = \frac{{d_{30}^{2} }}{{d_{60} \cdot d_{10} }}} \hfill \\ \end{array} } \right.$$

d_10_, d_30,_ and d_60_ represent the corresponding particle sizes when the mass smaller than the particle size accounts for 10%, 30%, and 60% of the total mass, respectively. The linear interpolation method can be used to determine d_10_, d_30_, and d_60_. This is an approximate calculation method that uses the proportional relationship between independent variables to solve unknown functions. If $X\left( {A_{1} ,B_{1} } \right)$ and $Y\left( {A_{2} ,B_{2} } \right)$ are two points, the point $C\left( {A,B} \right)$ is on the straight line of the above two points, that is, C is between points X and Y.

The values in the gradation curve were brought into Eq. (1), and the values of d_10_, d_30_, d_60_, C_u_, and C_c_ obtained are shown in Table 2.

**Table 2:** Values of coefficient of uniformity and coefficient of curvature.

Specimen size	d_10_	d_30_	d_60_	C_u_	C_c_
0.5–1 mm	0.79	0.93	1.10	1.39	1.00
1–2 mm	1.46	1.71	1.96	1.34	1.02
2–5 mm	2.97	3.40	3.90	1.31	0.91
5–10 mm	5.43	6.34	7.50	1.38	0.99
10–15 mm	12.06	13.42	14.75	1.22	1.01
15–20 mm	15.22	16.49	18.22	1.20	0.98

It can be seen from Table 2 that the C_u_ value was much smaller than 5, and the C_c_ value was close to 1. Therefore, the particle composition was considered to be relatively uniform and continuous. In the comparison test of the concrete specimens from the above six kinds of particles, the propagation characteristics and attenuation law of elastic waves were mainly affected by these aggregate particles, which achieved the expected experimental purpose.

## Analysis

### Amplitude attenuation law

**Figure 4:**
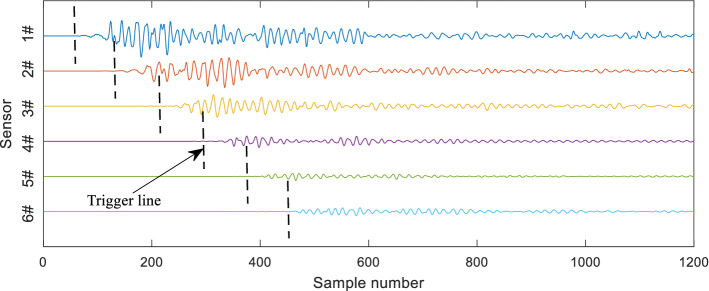
Signals of lead-break testing of 0.5–1 mm samples.

The propagation process of the elastic waves generated by the lead-breaking test on the concrete specimen indicated that the attenuation of the characteristic parameters of amplitude, energy, and frequency was the result of the combined effect of the strength of the sound source and the physical and mechanical properties of the concrete medium. This test was repeated four times for each specimen in the same way. The signals monitored by the six sensors are shown in Fig. 4. In this paper, the threshold method is used to identify whether the signal arrives, that is, when the amplitude exceeds the threshold for the first time, it can be determined as a trigger line. The black dotted line in the figure was the threshold trigger line. The elastic wave reached the 1–6# sensors in sequence according to the propagation distance, and the waveform amplitude gradually decreased; however, the rate of decrease in amplitude was different (i.e., the attenuation of the signal amplitude in the coarse aggregate specimen was relatively more rapid).

Since concrete material is an incomplete elastic body, fluctuating energy loss occurs when stress waves propagate through it. According to the stress wave propagation attenuation theory and the quality factor Q theory^15^, the change in the amplitude of the AE wave during propagation can be expressed as:

2 $$A(x) = A_{0} \exp \left( { - \frac{\pi f}{{VQ}}x} \right) = A_{0} \exp \left( { - \alpha x} \right)$$

where $A\left( x \right)$ is the amplitude of the AE propagation distance x, dB;

$A_{0}$ is the initial amplitude of the AE signal, dB; $f$ is the frequency of the AE signal, Hz; $x$ is the propagation distance of the AE signal, m; $V$ is the propagation speed of the AE signal, m/s; $Q$ is the quality factor of the rock mass medium; and $\alpha$ is the attenuation coefficient of the AE signal.

**Figure 5:**
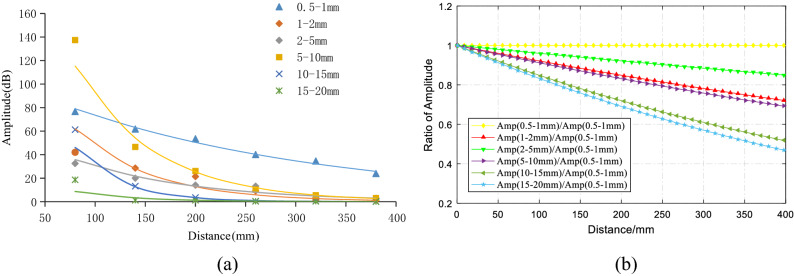
Attenuation of amplitude with particle sizes. (**a**) Attenuation of amplitude. (**b**) Relative attenuation of amplitude.

It can be seen that the amplitude of the elastic wave signal decayed with a negative exponential function as the propagation distance increased, and the attenuation rate depended on the attenuation coefficient $\alpha$ in Eq. (2). To analyze the law of elastic wave amplitude attenuation, the amplitude peaks were obtained by filtering and spectrum conversion, and the average of all four tests results for each particle size was obtained. The results of the fitting of the average amplitude of the six groups of experiments are shown in Fig. 5a. The fitting coefficients of the six specimens were 0.9829, 0.9197, 0.9153, 0.9901, 0.9545, and 0.8744, which can be seen in the confidence interval at 95%, and the attenuation of amplitude with distance confirmed a good negative exponential correlation. After fitting, the elastic wave amplitude attenuation coefficients of the 0.5–1 mm, 1–2 mm, 2–5 mm, 5–10 mm, 10–15 mm, and 15–20 mm specimens were 0.004, 0.120, 0.008, 0.130, 0.180, and 0.200; and there was a good correlation between the particle size of the aggregate and the attenuation coefficient of the amplitude (except for the 1–2 mm specimen, which will be explained in Table 3). The attenuation coefficient of the elastic wave amplitude in the concrete varied with the particle size (i.e., the larger the aggregate particle size, the faster the attenuation rate of the elastic wave amplitude). To further illustrate the influence of the aggregate particle size on amplitude attenuation, the fitting curves of the different particle sizes were divided by the fitting curves of the 0.5–1 mm specimens and normalized. The results are shown in Fig. 5b and clearly show that the larger the aggregate the particle size was, the faster the relative velocity of the elastic wave amplitude attenuation.

**Table 3:** Quality factors of samples with different particle sizes.

Particle size	Quality parameters			
	$E$ (J/Hz)	$\Delta E$ (J/Hz)	$\Delta E/E$	$Q$
0.5–1 mm	54.13	41.23	0.76	8.25
1–2 mm	36.51	34.01	0.93	6.74
2–5 mm	26.28	23.95	0.91	6.89
5–10 mm	13.86	13.55	0.98	6.43
10–15 mm	14.07	14.03	1.00	6.30
15–20 mm	14.25	14.23	1.00	6.29

In this table, E is the energy spectral density of sensor 1#, and $\Delta E$ is the energy spectral density loss of elastic wave from sensor 1# to sensor 6#.

### Spectrum evolution law

**Figure 6:**
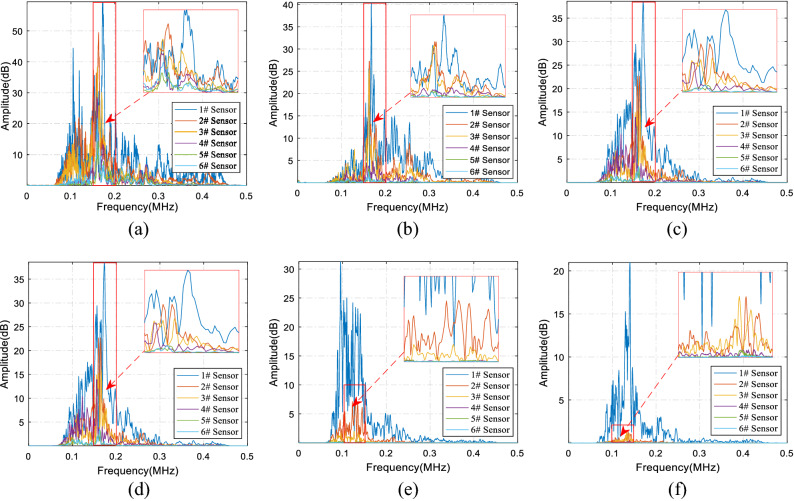
The spectral distribution of 0.5–1 mm (**a**), 1–2 mm (**b**), 2–5 mm (**c**), 5–10 mm (**d**), 10–15 mm (**e**), and 15–20 mm (**f**) samples.

In theory, the signals emitted by AE sources contain information that reflects their essential characteristics. Spectrum analysis is complementary to time-domain signals, with the belief that information that cannot be found in time-domain signals can be reflected in the frequency domain. Therefore, the evolution law of elastic wave spectrum in concrete specimens of different particle sizes was analyzed, and Fast Fourier Transform (FFT) was used to obtain the frequency domain information of the signal. The spectrum distribution of each specimen is shown in Fig. 6a–f. The results were as follows. (1) The amplitude of the 1# sensor signal was the largest in the spectrum of all the samples, and the amplitude of the 2–6# sensor signals decreased sequentially, indicating that with the propagation of the elastic wave, the signal strength of each frequency band gradually decreased. (2) The signal attenuation rate in each particle size was different. The sensor signals of the smaller aggregate concrete specimens (e.g. 0.5–1 mm, 1–2 mm) were superimposed on each other, and the attenuation was not obvious. Comparing the signals of sensors 1–6#, the attenuation of amplitude is more obvious in concrete samples with larger aggregate (e.g. 10–15 mm, 15–20 mm). (3) Although all the tests used very similar lead-breaking tests, the signal frequency distribution obtained in the test was very different. In the 0.5–1 mm and 1–2 mm samples, the proportion of the high frequencies above 300 kHz was relatively large; but in the 2–5 mm and 5–10 mm specimens, the proportion gradually decreased. Furthermore, in the 10–15 mm and 15–20 mm specimens, the high-frequency ratio became very low.

**Figure 7:**
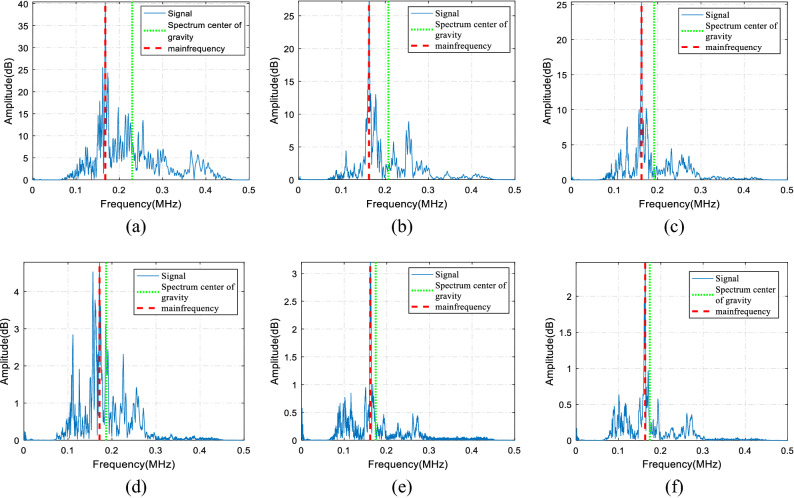
Spectrum distribution of the 1–6# sensors (**a**–**f**) of 1–2 mm sample.

Thereafter, the frequency spectrum evolution law of the elastic wave with the propagation process in the concrete sample of the same aggregate size was analyzed. The spectrum distribution of the different sensor signals is shown in Fig. 7a–f. The results show that, as the elastic waves propagated, the high-frequency signals of 200 kHz and above exhibited very large attenuation. The frequencies were mainly distributed at 100–300 kHz of sensors 1# and 2#; the attenuation of high-frequency signals at 300 kHz and above was very significant. The signal frequency distribution of the 3# sensor was mainly concentrated in the range of 100–200 kHz while the frequency signal of 200–300 kHz dropped sharply. At this time, the signal frequency distribution gradually approached the low-frequency area, and the relative proportion of the high-frequency signal decreased significantly. In the 5# and 6# sensors, the distribution of the high-frequency signals was much lower than that of the low-frequency signals, and the amplitude of the frequency signals above 300 kHz was negligible. To summarize the above analysis, the higher the frequency was in the same concrete specimen, the faster the attenuation, and vice versa.

The above qualitative analysis addressed the attenuation rate of signals in different frequency bands. To quantify the evolution trend of the frequency of the AE signals, the main frequency and the center of gravity of the spectrum were selected to represent the frequency attenuation law. The red dotted line in Fig. 7 represents the main frequency (peak frequency, i.e. the frequency corresponding to the amplitude peak), and the green dotted line represents the center of gravity of the spectrum. The main frequency values from the 1–6# sensors were 0.1676 MHz, 0.1627 MHz, 0.1624 MHz, 0.1718 MHz, 0.1613 MHz, and 0.1614 MHz.

The main frequency of the spectrogram only represents the amplitude peak, and its analysis cannot represent all the propagation characteristics of signals composed of different frequencies. It does not have a universal law, and it cannot be used to describe the attenuation law of the signal as a whole. The frequency with the same area on both sides of the amplitude and frequency represents the center of gravity of the spectrum. The center of gravity of the spectrum combines the signals of different frequencies and amplitude, which are more comprehensive and representative, and also can represent the propagation characteristics of the overall signal. The spectrum weight of the 1–6# sensors was 0.2297 MHz, 0.2074 MHz, 0.1917 MHz, 0.1868 MHz, 0.1740 MHz, and 0.1899 MHz, respectively. It can be seen that the spectral center of gravity of the different sensors was near linearly attenuated and can well explain the attenuation law of AE signals under different aggregate size parameters.

**Figure 8:**
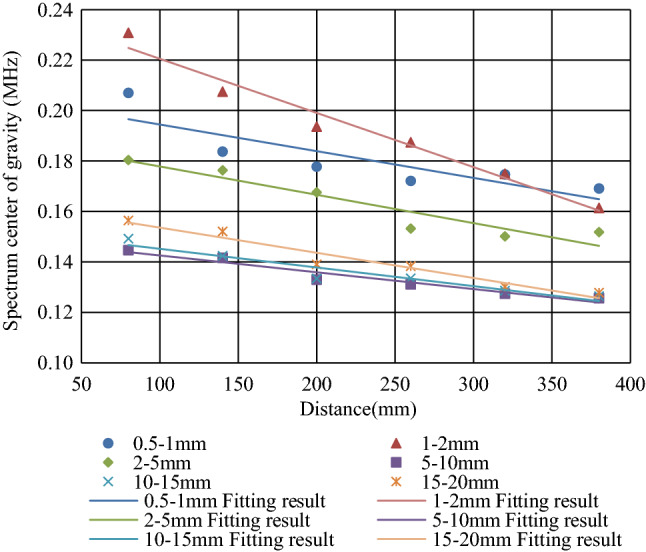
Attenuation law of spectrum center of gravity.

To further explore the attenuation of the center of gravity of the spectrum in concrete with different particle sizes, the four test data of each specimen were averaged and fitted. The results are shown in Fig. 8. Through linear fitting, the attenuation coefficients of 0.5–1 mm, 1–2 mm, 2–5 mm, 5–10 mm, 10–15 mm, and 15–20 mm were 0.0001, 0.0002, 0.0001, 0.00007, 0.00007, and 0.0001, respectively. It can be seen that the spectral center of gravity of the AE signal was linearly attenuated and the size of particle size had no obvious effect on the attenuation rate of the spectral center of gravity.

### Energy attenuation law

Energy is a characteristic parameter that comprehensively reflects the strength of the AE signal. In this study, the energy spectrum was used instead of the energy parameter, which reflects the relationship between energy and signal frequency. Based on Parseval's theorem, the AE signal satisfied the following equation^13^:

3 $$E = \int_{{{ - }\infty }}^{\infty } {f^{2} } \left( t \right)dt = \int_{ - \infty }^{\infty } {\left| {\Phi \left( \omega \right)} \right|}^{2} d\omega$$

Among them, E represents the signal energy, $f(t)$ represents the AE signal, $t$ represents the time, $\omega$ represents the frequency, and $\Phi \left( \omega \right)$ represents the function of the AE signal after Fourier transform. Equation (3) can be understood if the AE energy is equal to the integral of $\Phi \left( \omega \right)$ in the frequency domain, then $\Phi \left( \omega \right)$ can be called the energy spectrum (density).

**Figure 9:**
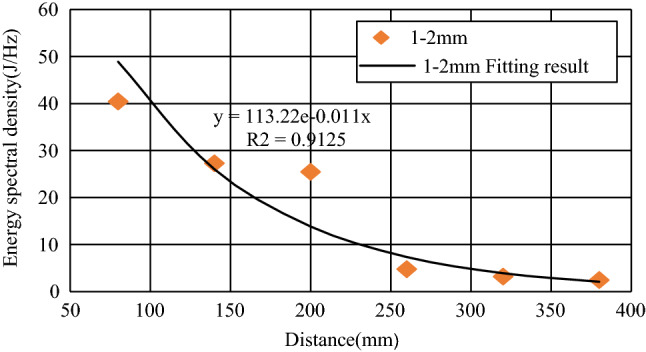
Energy spectrum density attenuation law of 1–2 mm sample.

Because the energy of the AE signal of each test was inconsistent, this paper shows Test 1 of the 1–2 mm specimen as an example to make the energy spectrum density map of the 1–6# sensors. The energy spectrum peaks were 40.4253 J/Hz, 27.2860 J/Hz, 25.4754 J/Hz, 4.7795 J/Hz, 3.2150 J/Hz, and 2.4745 J/Hz. The analysis found that the peak of the propagation energy spectrum of the elastic wave showed a negative exponential decay trend. The fitting result is shown in Fig. 9. The attenuation law reflected by the energy spectrum density diagram is similar to the spectrum attenuation. This shows that the energy can be used as an important index of response signal attenuation.

**Figure 10:**
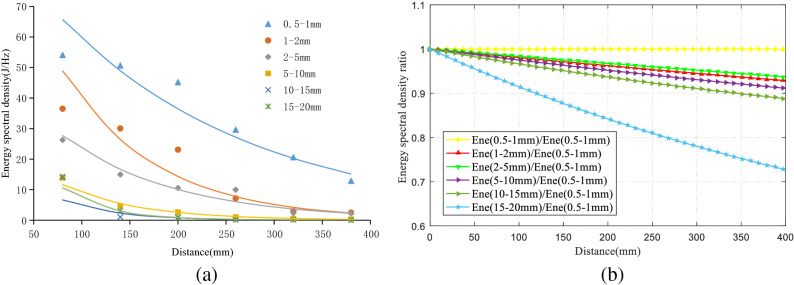
Law of attenuation of energy spectrum density. (**a**) Energy spectral density attenuation comparison. (**b**) Relative attenuation of energy spectral density.

To study the law of peak attenuation of the energy spectrum of different particle sizes, the average energy spectrum peak value of the six groups’ tests was determined, and the law of peak attenuation of energy spectrum was obtained by fitting, as shown in Fig. 10a to be 0.5–1 mm, 1–2 mm, 2–5 mm, 5–10 mm, 10–15 mm, and 15–20 mm particle size elastic wave energy spectrum peak attenuation coefficients were 0.005, 0.010, 0.008, 0.013, 0.018, and 0.021, respectively. It can be seen that, except for the 1–2 mm specimen, the larger the aggregate particle size was, the faster the attenuation rate of the elastic wave energy spectrum peak. The fitting curves of the different particle diameters were divided by the fitting curves of the 0.5–1 mm specimens, respectively, and were normalized. The results are shown in Fig. 10b. It can be seen that the larger the aggregate particle size was, the faster the relative velocity of the elastic wave energy spectrum peak attenuation.

The quality factor, Q, was used to describe the absorption characteristics of the elastic wave energy of the rock. The smaller the Q value was, the greater the elastic wave energy loss and the faster the attenuation, and vice versa. The quality factor Q is defined here as the reciprocal of the ratio of the energy $\Delta E$ lost by vibration to the total energy $E$ in a period (within a wavelength distance)^15^, which is:

4 $$\frac{2\pi }{Q} = \frac{\Delta E}{E}$$

In this formula, $E$ is the elastic energy under the state of maximum stress and strain, and $\Delta E$ is the energy loss of one cycle of elastic wave vibration.

Only the characteristic parameters of the signals of the 1–6# sensors were known, and the energy of the AE source was unknown. The 1# sensor was the closest to the AE source so the energy of the 1# sensor was approximately used as the energy of the AE source. The quality factor Q of each particle size specimen was calculated according to the definition formula of quality factor Q as shown in Table 3. It was found that if Q_2–5 mm_ > Q_1–2 mm_, then the amplitude and energy spectrum peak decay rate of the 1–2 mm sample was greater than the 2–5 mm sample, as shown in Fig. 5 and Fig. 10.

**Figure 11:**
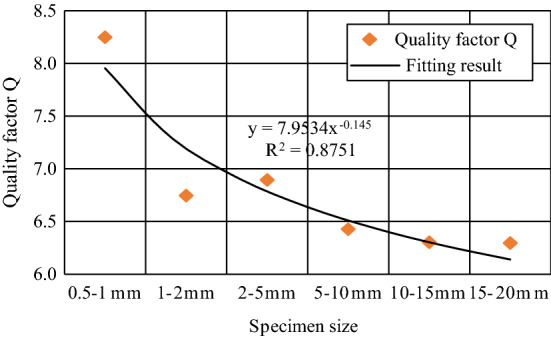
Attenuation law of quality factor of samples with different particle sizes.

From the results of fitting the quality factor, as shown in Fig. 11, it can be seen that the quality factor Q value decreases with the increase of aggregate particle size (i.e., the larger the concrete aggregate particle size was, the smaller the quality factor Q and the faster the relative amplitude and energy decay rate).

### Ring count evolution law

**Figure 12:**
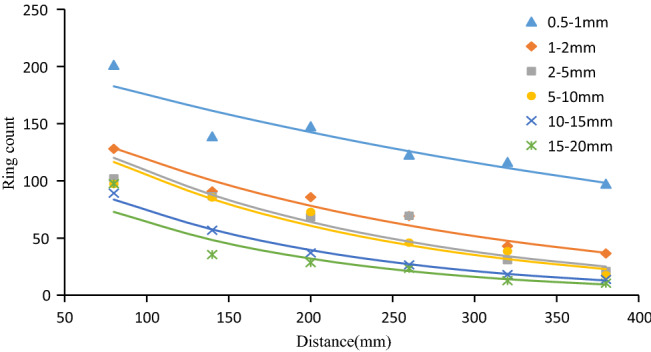
The ring count attenuation with different particle sizes.

The ring count refers to the number of oscillations of the AE signal exceeding the threshold^7^. To explore the attenuation law of ring counts with different particle sizes, the average value of six groups of ring counts was curve-fitted, and the results are shown in Fig. 12. Through fitting, it was found that the ring count was also attenuated with a negative exponential function with an increase in the elastic wave propagation distance. The attenuation coefficients were 0.002, 0.004, 0.005, 0.005, 0.005, and 0.007, which indicated an increasing trend. It also showed that the particle size of the aggregate had a small effect on the attenuation of the ring count. For the specimens with larger aggregate size, the ring count attenuation rate was relatively larger.

**Figure 13:**
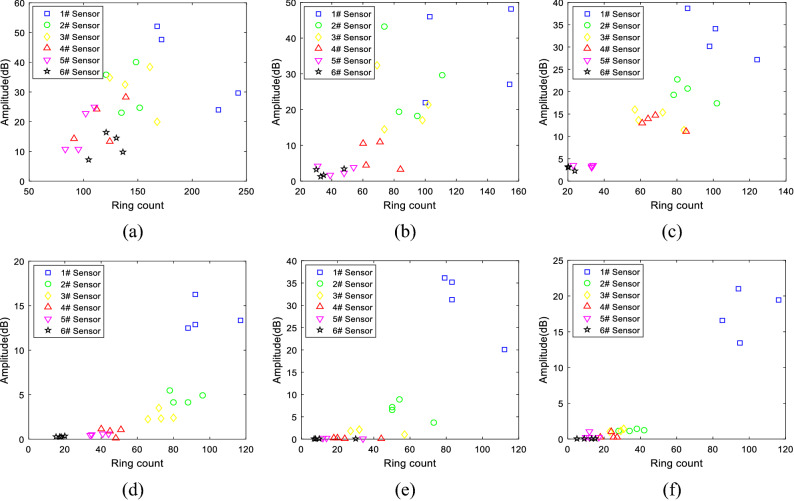
Correlation diagram of ring count and amplitude of 0.5–1 mm (**a**), 1–2 mm (**b**), 2–5 mm (**c**), 5–10 mm (**d**), 10–15 mm (**e**), and 15–20 mm (**f**) samples .

Both the amplitude and the ring count are important AE characteristic parameters, and the ring count is closely related to the amplitude of the signal. As the elastic wave propagates, the signal amplitude gradually attenuates, and the number of oscillations that cross the threshold also decay gradually, and the corresponding ring count also decreases. The relationships between the ring count and amplitude of all six different particle size samples were found as shown in Fig. 13a–f. It can be seen from the figure that as the elastic wave propagated, the ring count was positively correlated with the amplitude (i.e., the larger the ring count was, the greater the amplitude). The smaller the aggregate particle size (such as 0.5–1 mm, 1–2 mm) was, the greater the dispersion of the feature vector in the amplitude-ring count feature space, as shown in Fig. 13a, b. On the contrary, the distribution of the feature vectors that corresponded to the samples with large aggregate particle sizes was relatively concentrated, as shown in Fig. 13c–f. In addition, in this feature space, as the propagation distance increased, the feature vector gradually moved to the lower-left corner.

### Rise time evolution law

**Figure 14:**
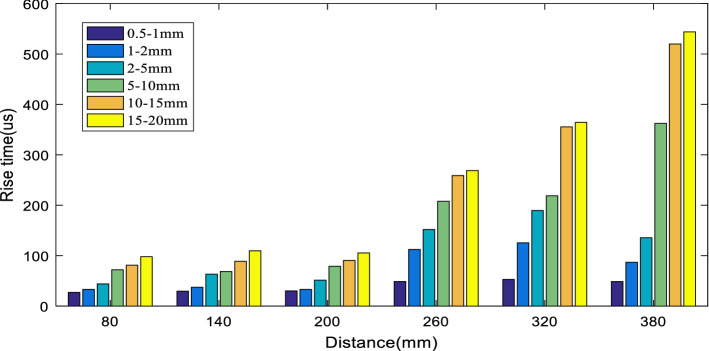
Analysis of the rise time of samples with different particle sizes.

Rise time is the time that the AE signal crosses the threshold for the first time to reach the highest amplitude value. A histogram of the rise time of the six groups of different particle size concrete samples is shown in Fig. 14. It can be seen from the figure that with the propagation of elastic waves, the rise time demonstrates a significant upward trend. In rocky media, within a certain frequency range, the higher the frequency, the faster the wave propagation speed^13^. Due to the rapid attenuation of high-frequency signals, the AE wave propagation speed may be reduced macroscopically. The longitudinal wave triggers the threshold, and the shear wave determines the maximum value to a large extent. The difference between the two is greater at a distance from the sound source, which may lead to an increase in rise time.

**Figure 15:**
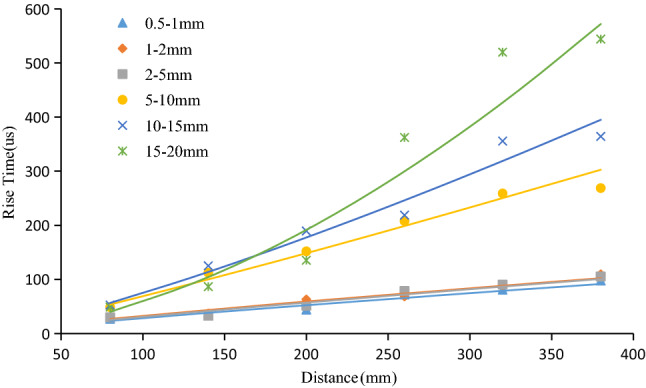
Evolution rule of rise time of samples with different particle sizes.

To further explore the influence of aggregate particle size on the signal rise time, the average value of the rise time of the six different particle size specimens was taken and fitted with a power function ($y = ax^{b}$). The results are shown in Fig. 15. The consistency between the rise time of the six samples and the fitting curve are all above 0.9, indicating that the fitting curve had high confidence. It can be seen from the formula of the fitting curve that the rise time coefficients b of the six particle size samples were 0.8724, 0.8854, 0.8854, 1.1086, 1.2454, and 1.7032, and the growth rate of the rise time was positively correlated with the aggregate particle size. In addition, the larger the concrete aggregate particle size was, the faster the signal rise time increased.

**Figure 16:**
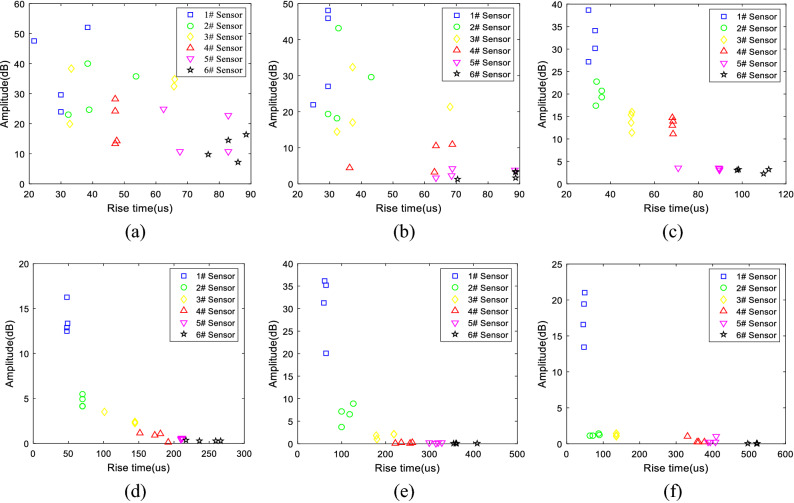
Correlation diagram of rise time and amplitude of 0.5–1 mm (**a**), 1–2 mm (**b**), 2–5 mm (**c**), 5–10 mm (**d**), 10–15 mm (**e**), and 15–20 mm (**f**) samples.

The rise time and amplitude were put into the feature space, and a correlation diagram of all the six groups of particle size samples was drawn as shown in Fig. 16a–f. It can be seen from the figure that with the propagation of elastic waves, the rise time was negatively correlated with the amplitude (i.e., the larger the rise time was, the smaller the amplitude). Also, the smaller the aggregate particle size was (e.g., 0.5–1 mm, 1–2 mm), the greater the dispersion of the feature vector in the amplitude-ring counting feature space, as shown in Fig. 16a, b. Otherwise, the distribution of the feature vectors corresponding to the samples with large particle sizes was relatively concentrated, as shown in Fig. 16c–f. In addition, in the feature space of the rise time and amplitude, as the propagation distance increased, the feature vector gradually moved to the lower right corner.

## Discussion

The random distribution of aggregates in the concrete block leads to a certain degree of non-uniformity and anisotropy of the material, which makes the acoustic emission monitoring data in this study have greater dispersion. However, the statistical data of the experimental results is sufficient to show that the impact of concrete aggregate particle size on the attenuation of elastic waves is significant and regular.

In the related literature of acoustic emission research, Pencil lead breaks (Hsu-Nielsen sources) are widely used to simulate acoustic emission sources because of their ease of control. Part of the elastic wave propagates through the interior of the material, that is, the bulk wave and the other part mainly propagates through the interface, that is, the surface wave. The composition of the AE signal monitored by the ceramic piezoelectric sensor in this experiment is complex, including body waves and surface waves. Due to the limitations of test conditions and thickness of concrete specimens, it is difficult to distinguish between body waves and surface waves in the signal in this study. This is also an aspect that needs to be considered in the next study.

By studying the attenuation laws and characteristics of various elastic wave parameters in concrete materials, it can provide a basis for optimizing the accuracy of the acoustic emission source location. In the process of localization of the traditional acoustic emission source area, parameters such as amplitude and ringing count are generally regarded as a linear attenuation process and used to trace the location of the rupture point, but this is different from the real situation. Therefore, the nonlinear attenuation characteristics of AE parameters in this study are very important, which can provide a research basis for further optimization of source positioning.

## Conclusion

In this paper, six concrete specimens composed of six kinds of aggregates with different particle sizes were used to investigate the attenuation evolution law of elastic wave characteristic parameters with increasing propagation distance and the role of aggregate particle size in elastic wave attenuation. The main conclusions are as follows:

1. The analysis of the attenuation law of AE signal amplitude from the time domain revealed that the amplitude attenuation conformed to the negative exponential function, with attenuation coefficients of 0.004, 0.12, 0.008, 0.13, 0.18, and 0.20, and the attenuation rate of the elastic wave amplitude was positively correlated with the aggregate particle size.
2. The analysis of the frequency attenuation law of AE signal from the frequency domain found that the higher the signal frequency was, the faster the attenuation, and vice visa. The linear fitting of the signal spectrum center of gravity yields attenuation coefficients were 0.0001, 0.0002, 0.0001, 0.00007, 0.00007, and 0.0001. It can be seen that the aggregate particle size had little effect on the attenuation rate of the spectrum center of gravity.
3. Using the energy spectrum density instead of energy to analyze the attenuation law, it was found that the peak of the energy spectrum was attenuated by a negative exponential function. The attenuation coefficients were 0.005, 0.01, 0.008, 0.013, 0.018, and 0.021. The attenuation rate of the elastic wave energy spectrum peak was found to be positively correlated with the aggregate particle size, which was verified by the Q values.
4. Statistical analysis and curve-fitting on the ring count of each specimen were performed, which produced attenuation coefficients of 0.002, 0.004, 0.005, 0.005, 0.005, and 0.007. The increasing trend indicated that the aggregate particle size had a certain effect on the ring count. As a result, the aggregate particle size increased, and the ring count attenuation rate increased simultaneously. In addition, in the feature space of the ring count and amplitude, the ring count and amplitude had a positive correlation; and the larger the particle size of the aggregate was, the more the signal feature vector distribution was concentrated to the lower-left corner.
5. The statistical analysis performed on the ring count of each specimen produced attenuation coefficients of 0.002, 0.004, 0.005, 0.005, 0.005, and 0.007, which indicated that the aggregate particle size had a certain effect on the ring count. As the aggregate particle size increased, the ring count decay speed increased. In addition, the ring count and amplitude had a certain positive correlation, and the test data of the sample with a smaller particle size were more discrete.
6. The rise time coefficients *b* of the six particle size samples were 0.8724, 0.8854, 0.8854, 1.1086, 1.2454, and 1.7032. The larger the aggregate size was, the faster the rise time increased. In addition, in the feature space of rise time and amplitude, the rise time had a negative correlation with the amplitude, and the test data of the sample with a smaller particle size were more discrete.
